# Complex regulatory network allows *Myriophyllum aquaticum* to thrive under high-concentration ammonia toxicity

**DOI:** 10.1038/s41598-019-41236-8

**Published:** 2019-03-18

**Authors:** Rui Wang, Shengjun Xu, Haishu Sun, Shugeng Feng, Cancan Jiang, Sining Zhou, Shimin Wu, Guoqiang Zhuang, Baodong Chen, Zhihui Bai, Xuliang Zhuang

**Affiliations:** 1 Key Laboratory of Environmental Biotechnology, Research Center for Eco-Environmental Sciences, Chinese Academy of Sciences, Beijing, 100085 China; 2 College of Resources and Environment, University of Chinese Academy of Sciences, Beijing, 100049 China; 3 State Key Laboratory of Urban and Regional Ecology, Research Center for Eco-Environmental Sciences, Chinese Academy of Sciences, Beijing, 100085 China

## Abstract

Plants easily experience ammonia (NH_4_^+^) toxicity, especially aquatic plants. However, a unique wetland plant species, *Myriophyllum aquaticum*, can survive in livestock wastewater with more than 26 mM NH_4_^+^. In this study, the mechanisms of the *M. aquaticum* response to NH_4_^+^ toxicity were analysed with RNA-seq. Preliminary analysis of enzyme activities indicated that key enzymes involved in nitrogen metabolism were activated to assimilate toxic NH_4_^+^ into amino acids and proteins. In response to photosystem damage, *M. aquaticum* seemed to remobilize starch and cellulose for greater carbon and energy supplies to resist NH_4_^+^ toxicity. Antioxidative enzyme activity and the secondary metabolite content were significantly elevated for reactive oxygen species removal. Transcriptomic analyses also revealed that genes involved in diverse functions (e.g., nitrogen, carbon and secondary metabolisms) were highly responsive to NH_4_^+^ stress. These results suggested that a complex physiological and genetic regulatory network in *M. aquaticum* contributes to its NH_4_^+^ tolerance.

## Introduction

As a major nitrogen source, ammonia (NH_4_^+^) is important for plant growth and development in soil and freshwater ecosystems^1,2^. However, high concentrations of NH_4_^+^, as a kind of abiotic stress, can damage plant cells and cause a series of syndromes, such as leaf chlorosis, a decrease in net photosynthesis and rhizosphere acidification^2,3^. As reported in previous studies, abiotic stresses impair productivity, reducing average yields by more than 50% globally^4^. During the past several decades, a worldwide decline in submerged macrophytes also occurred in many eutrophic lakes, and a high concentration of NH_4_^+^ in the aquatic environment was regarded as the main cause^1,5^. Therefore, NH_4_^+^ toxicity is a significant ecological and agricultural issue and an important phenomenon in cell biology^3^.

Plants have evolved various defense mechanisms at multiple levels in response to unfavourable environments including NH_4_^+^ stress^1,3^. In higher plants, once NH_4_^+^ has entered the cells, the glutamine synthetase/glutamate synthase (GS/GOGAT) cycle is the first step in NH_4_^+^ assimilation^6,7^. GS catalyses a reaction that incorporates NH_4_^+^ into glutamate and generates glutamine (Gln) as a product^8,9^. GOGAT transfers the amine group in the amide side chain of Gln to 2-oxoglutarate (2-OG), yielding two molecules of glutamate; one molecule serves as a substrate for GS, whilst the other is used for further metabolism^5,8^. NH_4_^+^ assimilation is closely linked to carbon metabolism since the supply of organic acids, which is maintained by the tricarboxylic acid (TCA) cycle, is indispensable in amino acid synthesis^10^. Thus, a high availability of carbon skeletons is essential for NH_4_^+^ assimilation in plant cells. Moreover, accelerated decomposition of starch is also found in plant cells under abiotic stress (e.g., cold, salt and drought) and provides more carbon skeletons and energy for abiotic stress protection^11,12^. However, metabolism acceleration produces redundant reactive oxygen species (ROS) and disrupts internal ROS homeostasis, thereafter leading to secondary oxidative stress and intracellular damage in plants^1,13^. Typically, plant cells upregulate the activity of antioxidant enzymes, such as peroxidase (POD), superoxide dismutase (SOD) and catalase (CAT), to remove excessive ROS^14^. Nevertheless, as an assistant antioxidant pathway, phenylpropanoid biosynthesis in plants provides substrates for the synthesis of many secondary metabolites, such as phenolics, flavonoids and lignin. These compounds are critical for defence responses to biotic and abiotic stresses^15–17^. However, these results were mainly obtained from terrestrial plants, and less is known about the mechanisms of NH_4_^+^ tolerance in wetland (aquatic) plant species.

Normally, the concentrations of NH_4_^+^ in natural waters such as rivers and lakes are no more than 14 uM. However, with the input of urban waste water and runoff from farmlands, the concentrations of NH_4_^+^ could significantly increase and even up to 26 mM in some constructed wetlands^18–20^. As aquatic plants, most wetland species are sensitive to high concentrations of NH_4_^+^ and are likely to experience NH_4_^+^ toxicity, which may seriously hinder their growth and ability to remove nutrients^16,20^. The reported symptoms of NH_4_^+^ toxicity range widely, and generally appear with external NH_4_^+^ concentrations above 0.1 to 0.5 mM^1,5^. For example, the growth rate of *Azolla filiculoides* decreased when the NH_4_^+^ concentration was above 0.1 mM, and root damage occurred when the NH_4_^+^ concentration was higher than 1 mM^21^. *Ceratophyllum demersum* cannot grow well when NH_4_^+^ concentrations were higher than 0.4 mM^22^. *Wolffia arrhiza* exhibited higher tolerance to NH_4_^+^ up to 4 mM^23^. However, *Myriophyllum aquaticum*, an important wetland plant species for biomass accumulation and nutrient removal^18,24^, is entirely different from *Myriophyllum spicatum*, which is used in ecological engineering for aquatic ecosystem restoration and is sensitive to NH_4_^+ ^^25^. *M. aquaticum* shows strong potential to resist NH_4_^+^ toxicity since it can grow in constructed wetlands with high concentrations of NH_4_^+^ (up to 26 mM)^24–26^. For coping with NH_4_^+^ toxicity, *M. aquaticum* has a specialized pathway that can achieve detoxification, and this pathway is highly distinct from that in sensitive species^5^. For example, *M. aquaticum* was found to convert accumulated NH_4_^+^ nitrogen into nitrate internally to avoid toxicity caused by the accumulation of NH_4_^+^ in tissues. Furthermore, the detoxification reaction was found to be closely related to high-quality asparagine synthetase (AS) and GS activities^5^. In other ways, *M. aquaticum* can elevate SOD and POD activities to remove redundant ROS and avoid oxidative damage^27^. However, these studies mainly focused on the leaves of *M. aquaticum*, and the functions of other key enzymes involved in nitrogen, carbon and antioxidative pathways have not been explored. Furthermore, gene regulation associated with NH_4_^+^ tolerance in *M. aquaticum* is still unknown and thus needs to be further explored.

Within this context, the present work hypothesized that NH_4_^+^ detoxification depends on network regulation in *M. aquaticum* including nitrogen, carbon and secondary metabolisms in roots and leaves. To test this hypothesis, we grew *M. aquaticum* under two concentrations of NH_4_^+^ (12 and 36 mM) that suppress *M. aquaticum* growth^27^ for 14 days. These two groups were defined as A12 and A36 group with a negative control group (NC group) under 1 mM NH_4_^+^. An integrative approach was applied, first measuring plant biomass (defined as the growth rate) and NH_4_^+^ content and then determining nitrogen, carbon and antioxidative metabolites and enzyme activities. Finally, a transcriptomic analysis of roots and leaves was carried out to investigate the complexity of the molecular events underlying the response to NH_4_^+^ in this species. Gene Ontology (GO) enrichment and Kyoto Encyclopedia of Genes and Genomes (KEGG) pathway enrichment were used to identify the important biological processes operating in *M. aquaticum* roots and leaves.

## Results

### Responses to NH_4_^+^ stress

#### NH_4_^+^ content

**Figure 1:**
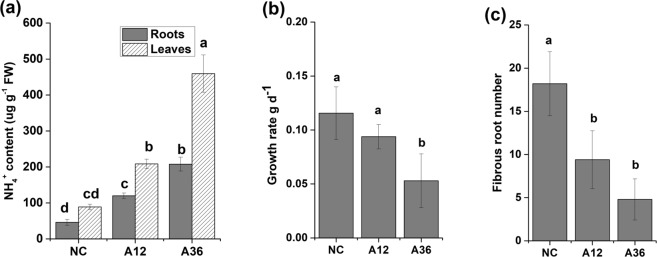
NH_4_^+^ content and growth characteristics of *Myriophyllum aquaticum*. (**a**) NH_4_^+^ content in roots and leaves. (**b**) Growth rate. (**c**) Fibrous root number. The values are the means ± SDs (n = 5). Different letters indicate a significant difference at *p* < 0.05.

Roots and leaves are important plant organs for nutrient absorption and energy assimilation, respectively. As the NH_4_^+^ concentration in the nutrient solution increased, the NH_4_^+^ content in the leaves of *M. aquaticum* increased significantly (*p* < 0.05) from 69 µg g^−1^ fresh weight (FW) in the NC group to 162 µg g^−1^ FW in the A12 group and 357 µg g^−1^ FW in the A36 group (Fig. 1a). Furthermore, the NH_4_^+^ content in roots also significantly increased in the NH_4_^+^-stressed groups. Interestingly, the NH_4_^+^ content in the leaves of each group was always higher than that in the roots, which indicated that most of the NH4^+^ was transported to the leaves.

#### Plant growth

Biomass reduction provoked by NH_4_^+^ toxicity is generally associated with the accumulation of NH_4_^+^ in tissues of different plant species^6,10^. With increased NH_4_^+^ content, the growth rate significantly decreased by more than 50%, from 0.116 g d^−1^ in the NC group to 0.053 g d^−1^ in the A36 group (Fig. 1b). Further, the number of fibrous roots also significantly decreased in the A12 and A36 groups (Fig. 1c). Spearman’s correlation analysis indicated that the changes in growth rate and fibrous root number were significantly correlated (*p* < 0.01) with the increases in NH_4_^+^ content in roots and leaves (Supplementary Table S1).

### Chlorophyll (Chl) and maximum quantum yield of photosystem II (Fv/Fm)

Energy metabolism also seems to be damaged by high concentrations of NH_4_^+ ^^28^. Photosynthetic pigment content (such as the Chl a and Chl b contents) decreased significantly (*p* < 0.05) in the A36 group compared to the NC group (Supplementary Fig. S1a). Moreover, Fv/Fm also significantly decreased from 0.830 in the NC group to 0.796 in the A36 group (Supplementary Fig. S1b). However, there were no significant differences in carotenoid (Car) among the different groups.

### Hydrogen peroxide (H_2_O_2_), superoxide anion radical (O_2_^−^) and malondialdehyde (MDA)

With the increase in NH_4_^+^ content, large amounts of H_2_O_2_ and O^2−^ were produced in *M. aquaticum* roots and leaves (Supplementary Fig. S2a,b) and easily accumulated in the leaves and roots, respectively. As a result of the increased NH_4_^+^ content, the MDA content also significantly (*p* < 0.05) increased in the A12 and A36 groups (Supplementary Fig. S2c). Spearman’s correlation analysis also showed that the MDA content was significantly correlated (*p* < 0.05) with the increased NH_4_^+^ content in roots and leaves.

To better understand the mechanism used by roots and leaves facing NH_4_^+^ stress, we further explored their transcriptomic and metabolic responses.

### De novo assembly and annotation of the root and leaf transcriptomes

#### Sequencing and assembling

As shown in Supplementary Table S2, a total of 131.48 G raw nucleotides (124.50 G clean nucleotides), equivalent to 941.20 million raw reads (914.70 million clean reads), were generated from 18 root and leaf samples belonging to the three groups (NC, A12 and A36). All clean reads of *M. aquaticum* were pooled together and then de novo assembled by Trinity. The transcriptome was de novo assembled to 97338 unigenes, with an N50 = 1187 bp.

#### Differential expression analyses under different concentrations of NH_4_^+^ stress

The transcriptomic changes of *M. aquaticum* roots and leaves in response to 12 and 36 mM NH_4_^+^ were analysed. The number of genes for which the expression was significantly (*p* < 0.05) altered based on different growth conditions was represented with Venn diagrams (Supplementary Fig. S3a,b). NH_4_^+^ treatment led to strong transcriptomic responses in which 4583 genes were differentially expressed in NH_4_^+^-stressed roots, while only 965 were differentially expressed in NH_4_^+^-stressed leaves. Under 12 mM NH_4_^+^, there were only 44 upregulated genes in the leaves (Supplementary Fig. S3b). Nevertheless, the number of upregulated genes in the A36 group was nearly 7-fold greater than that in the A12 group, reaching 351 in the former group, with little difference in the number of upregulated genes in the roots. Thus, different regulatory mechanisms in roots and leaves might have responded to the NH_4_^+^ stress.

#### GO enrichment analysis

**Figure 2:**
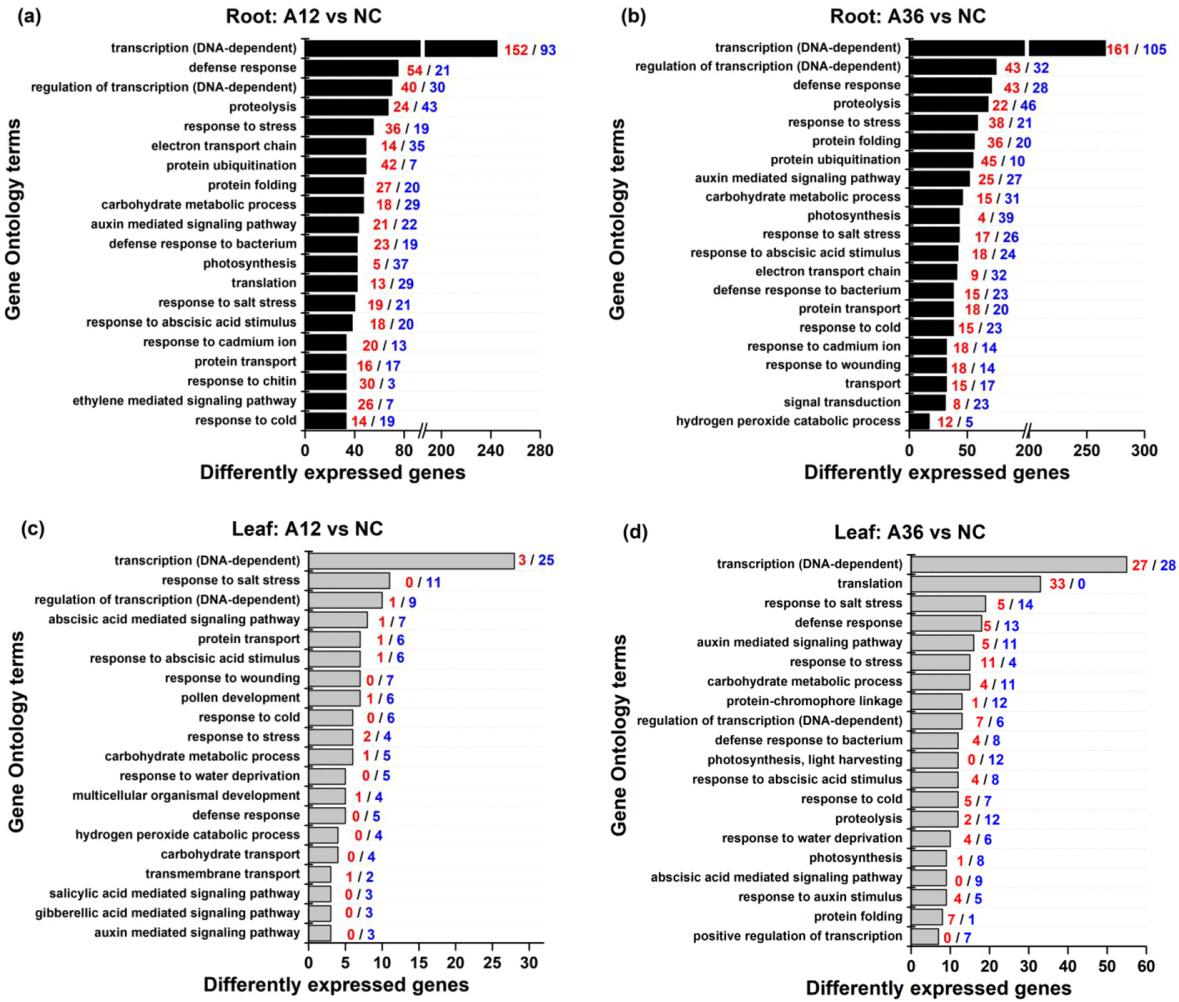
Gene Ontology (GO) analysis of significantly regulated genes in roots (**a**,**b**) and leaves (**c**,**d**) under 12 mM and 36 mM NH_4_^+^ treatments. Red indicates the number of upregulated genes. Blue indicates the number of downregulated genes.

GO analysis provided an overview of the processes most affected by NH_4_^+^ stress. The most enriched GO terms were similar in roots and leaves responding to 12 and 36 mM NH_4_^+^ (Fig. 2). However, there were large differences between the differentially expressed genes (DEGs) in terms of GO terms and the proportion of up- and downregulated genes. Genes related to stress response (e.g., salt, wounding, and cold stresses) were enriched in both roots and leaves under NH_4_^+^ stress, which indicated an increased response to NH_4_^+^ toxicity and a network response to different stress factors. Furthermore, genes for signalling regulation (e.g., auxin and abscisic acid in both roots and leaves and ethylene in roots) were also enriched, indicating that a complex signal regulation pathway responded to NH_4_^+^ stress^29^. Finally, the genes involved in light harvesting and photosynthesis were significantly (*p* < 0.05) downregulated in the leaves of the A36 group, indicating downsizing of the light-harvesting apparatus.

GO and KEGG analysis also indicated that strong transcriptional responses were observed for genes involved in processes such as NH_4_^+^ assimilation, amino acid synthesis, photosynthesis, starch and sucrose metabolism, ROS removal and phenylpropanoid biosynthesis, as discussed below. The responses of the most strongly regulated genes within these categories under NH_4_^+^ stress is shown in Supplementary Table S3. To verify the results of the RNA-seq analysis, qRT-PCR with specific primers was performed on eighteen selected genes involved in different metabolisms (Supplementary Table S4), and the results corresponded to those from RNA-seq (Supplementary Figs S4 and S5).

### Nitrogen metabolism

#### NH_4_^+^ assimilation

**Figure 3:**
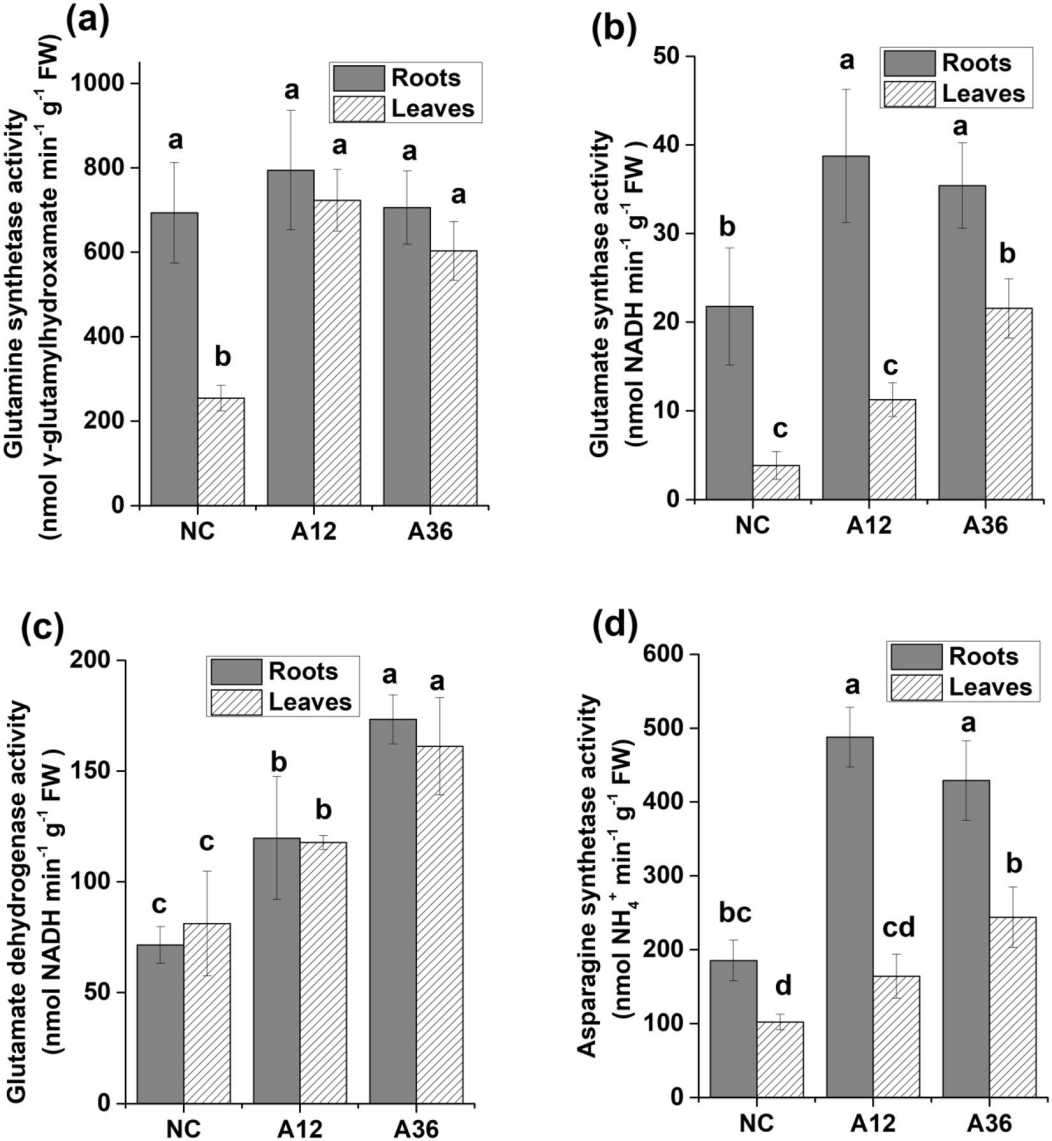
NH_4_^+^ assimilation-related enzyme activity in roots and leaves of *Myriophyllum aquaticum*. Enzyme activities were determined for glutamine synthetase (**a**), glutamate synthase (**b**), glutamate dehydrogenase (**c**), and asparagine synthetase (**d**). Values (means ± SDs) were determined from five biological replicates (n = 5). Different letters above the bars indicate a significant difference at *p* < 0.05.

**Figure 4:**
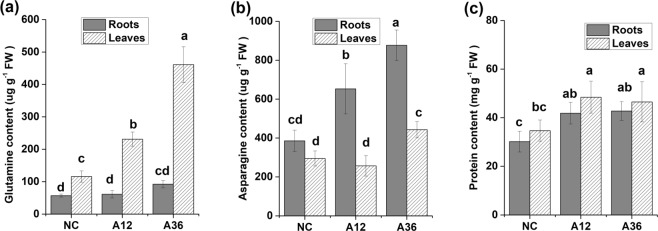
Effect of NH_4_^+^ toxicity on glutamine (**a**), asparagine (**b**) and protein content (**c**) in *Myriophyllum aquaticum* roots and leaves. Error bars represent the means ± SDs (n = 5). Different letters indicate a significant difference at *p* < 0.05.

With increased NH_4_^+^ content, significantly increased GS activity was observed in NH_4_^+^-stressed leaf cells (Fig. 3a). Interestingly, the GS in roots maintained a higher activity above 690 nmol γ-glutamyl hydroxamate (γ-GHA) min^−1^ g^−1^ FW than that in leaves in all groups. Correspondingly, the Gln content also significantly increased in the leaves under 12 mM NH_4_^+^, and the content doubled in the leaves of the A36 group compared with that of the NC group, although no significant increase was observed in the roots (Fig. 4a). As shown in Fig. 3b, increased NH_4_^+^ in the nutrient medium also caused significant differences in the GOGAT activity in the roots and leaves of *M. aquaticum* (*p* < 0.05). GOGAT activity reached a maximum value of 39 nmol NADH min^−1^ g^−1^ FW in the roots when plants were treated with 12 mM NH_4_^+^, while it peaked at 22 nmol NADH min^−1^ g^−1^ FW in the leaves when plants were treated with 36 mM NH_4_^+^. In addition, the gene encoding GOGAT was dramatically upregulated in NH_4_^+^-stressed root cells (Supplementary Table S3). Furthermore, the gene encoding GDH, another enzyme that catalyses the conversion of 2-OG to glutamate and the reverse, was also significantly upregulated in the roots. Simultaneously, the enzyme activity catalysing the conversion of 2-OG to glutamate was also significantly upregulated in both the roots and leaves of the A12 and A36 groups compared with the NC group (Fig. 3c). Finally, the activity of AS doubled in the roots of the A12 and A36 groups and in the leaves of the A36 group (Fig. 3d), although AS activity decreased slightly in the roots of the A36 group compared with the A12 group. Further, AS activity was significantly higher in the roots than in the leaves of each group, which indicated a faster rate of Asn synthesis in the roots than in the leaves. Correspondingly, the Asn content also increased significantly, reaching 877 µg g^−1^ FW in the roots of both the A12 and A36 groups, and increased to 443 µg g^−1^ FW in the leaves of the A36 group (Fig. 4b).

#### Biosynthesis of amino acids

**Figure 5:**
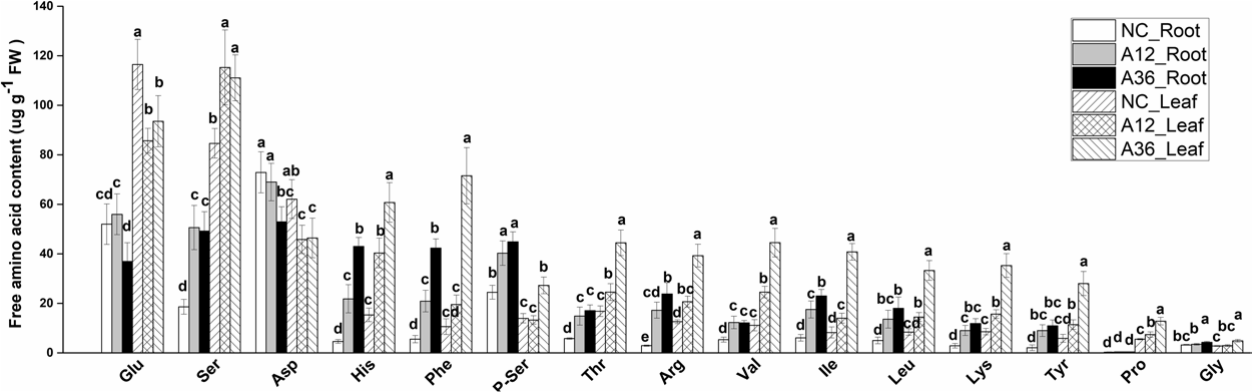
Effect of NH_4_^+^ on the free amino acid content in *Myriophyllum aquaticum* roots and leaves. Values (means ± SDs) were determined from five biological replicates (n = 5). Different letters above the bars indicate a significant difference at *p* < 0.05.

Changes in the fifteen most abundant free amino acids in response to NH_4_^+^ stress were quantified, and the results are shown in Fig. 5. The most abundant amino acid under normal conditions was glutamic acid (Glu), which accounted for 30% of the total free amino acid content in the leaves of *M. aquaticum*. However, serine (Ser) became the most abundant, accounting for 25% and 16% of the total free amino acid content in the leaves of the A12 and A36 groups, respectively. Phenylalanine (Phe) and tyrosine (Tyr), which are aromatic amino acids and substrates for phenol and flavonoid biosynthesis^15,16^, were also significantly increased in the roots and leaves under NH_4_^+^ stress. Interestingly, proline (Pro), an osmoticum and ROS scavenger often found to increase in response to NH_4_^+^ stress^30,31^, also showed a significant increase in the leaves but no significant change in the roots under NH_4_^+^ stress. Furthermore, the histidine, threonine, arginine, valine, isoleucine, leucine and lysine contents also increased significantly in response to NH_4_^+^ stress.

#### Protein biosynthesis

Protein, the main nitrogen-containing compound in plant cells, was measured in all groups. Protein synthesis seemed to be significantly accelerated in the tissues of the A12 and A36 groups, as the protein content was significantly higher in the A12 and A36 groups than in the NC group (Fig. 4c), which indicated that more free amino acids were used for protein synthesis under NH_4_^+^ stress.

### Carbon metabolism

#### Photosynthesis

As photosynthesis mainly occurs in leaves, we considered the gene regulation related to photosynthesis in leaves but not roots. In response to NH_4_^+^ stress, the expression levels of many genes encoding enzymes involved in light-harvesting and photosynthesis pathways were significantly repressed in the A36 group compared to the NC group (Supplementary Table S3), including four genes encoding light-harvesting complex (LHC) proteins in leaves. The downregulated expression of genes encoding Chl-binding proteins, Chl a and Chl b had fewer binding proteins to fix, which might have led to the significant decrease in the Chl pigment content and Fv/Fm (Supplementary Fig. S1a,b). Moreover, the expression of a gene encoding phosphoenolpyruvate carboxylase (PPC), which catalyses CO_2_ fixation, was 2.8-fold downregulated in the A36 group compared to the NC group, which indicated a reduced CO_2_ fixation efficiency in the leaves of the former group.

#### Starch cellulose and sucrose metabolism

**Figure 6:**
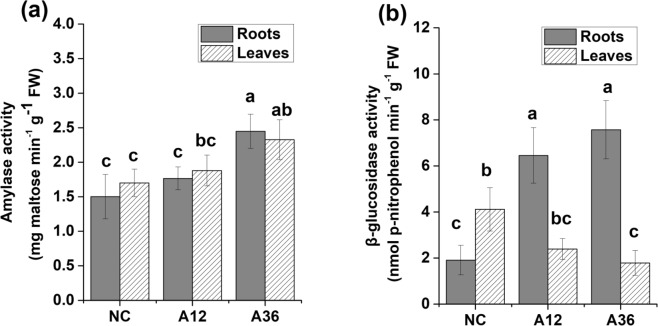
Activities of amylase (**a**) and β-glucosidase (**b**) under different NH_4_^+^ concentrations. Error bars represent the means ± SDs (n = 5). Different letters indicate a significant difference at *p* < 0.05.

In response to the reduced light energy and carbon acquisition efficiency in leaves, some genes associated with starch and cellulose decomposition were upregulated in NH_4_^+^-stressed cells. In particular, a gene encoding β-glucosidase was significantly upregulated 110.8-fold and 215.2-fold in the NH_4_^+^-stressed root cells of the A12 and A36 groups (Supplementary Table S3), respectively, indicating accelerated cellulose decomposition and glucose production for further utilization. Furthermore, a gene encoding β-amylase3 (BAM3) was upregulated more than 3.7-fold in the NH_4_^+^-stressed root cells in the A36 group. *M. aquaticum* seemed to upregulate a number of genes to decompose macromolecular organics under NH_4_^+^ stress. To verify this hypothesis, we subsequently measured the enzyme activity of amylase and β-glucosidase. The results revealed increased amylase activity in both the roots and leaves of the NH_4_^+^-stressed groups (Fig. 6a). Additionally, the β-glucosidase activity also significantly increased in the roots of the NH_4_^+^-stressed groups but decreased in the leaves of these groups (Fig. 6b). We further explored the amount of soluble carbohydrates that supply carbon skeletons for subsequent NH_4_^+^ assimilation into Gln and Asn and behave as signalling molecules. The amount of soluble carbohydrates significantly increased in the leaves of both the A12 and A36 groups but increased in the roots of only the A36 group (Supplementary Fig. S6a). Another significantly upregulated gene, namely, one encoding sucrose synthase, which catalyses sucrose synthesis from fructose and glucose, was observed in NH_4_^+^-stressed root cells. Correspondingly, a significant increase in sucrose content was observed in both the roots and leaves of the A36 group (Supplementary Fig. S6b). Additionally, the fructose content also significantly decreased in the NH_4_^+^-stressed groups (Supplementary Fig. S6c) as a result of fructose consumption for sucrose synthesis. Interestingly, the glucose content seemed to remain relatively stable in the NH_4_^+^-stressed groups (Supplementary Fig. S6d), even though the degradation of starch and cellulose was accelerated.

#### Decomposition of carbohydrate

KEGG analysis also indicated the promotion of several genes encoding enzymes potentially involved in glycolysis metabolism, pyruvate metabolism and the TCA cycle (Supplementary Table S3). Interestingly, significant upregulation of the gene encoding phosphoenolpyruvate carboxykinase (PckA) was observed in the roots, which indicated an accelerated transformation from oxaloacetate to phosphoenolpyruvate. Furthermore, the upregulation of pyruvate kinase (PK), which catalyses the transformation from phosphoenolpyruvate to pyruvate, seemed to increase the production of pyruvate, which was then transformed into acetyl-CoA for the TCA cycle. Several key genes connected to the TCA cycle were also upregulated in NH_4_^+^-stressed cells, such as the genes encoding citrate synthase (CS), aconitate hydratase (ACO) and adenosine triphosphate (ATP) citrate (pro-S)-lyase (ACLY). Interestingly, the gene encoding 2-OG dehydrogenase (OGDH), an important enzyme catalysing the oxidation of 2-OG and the synthesis of succinyl coenzyme A, was also upregulated in the roots and leaves under NH_4_^+^ stress. An accelerated TCA cycle could generate more ATP for plants to resist NH_4_^+^ toxicity.

### ROS removal and secondary metabolism

#### ROS removal

**Figure 7:**
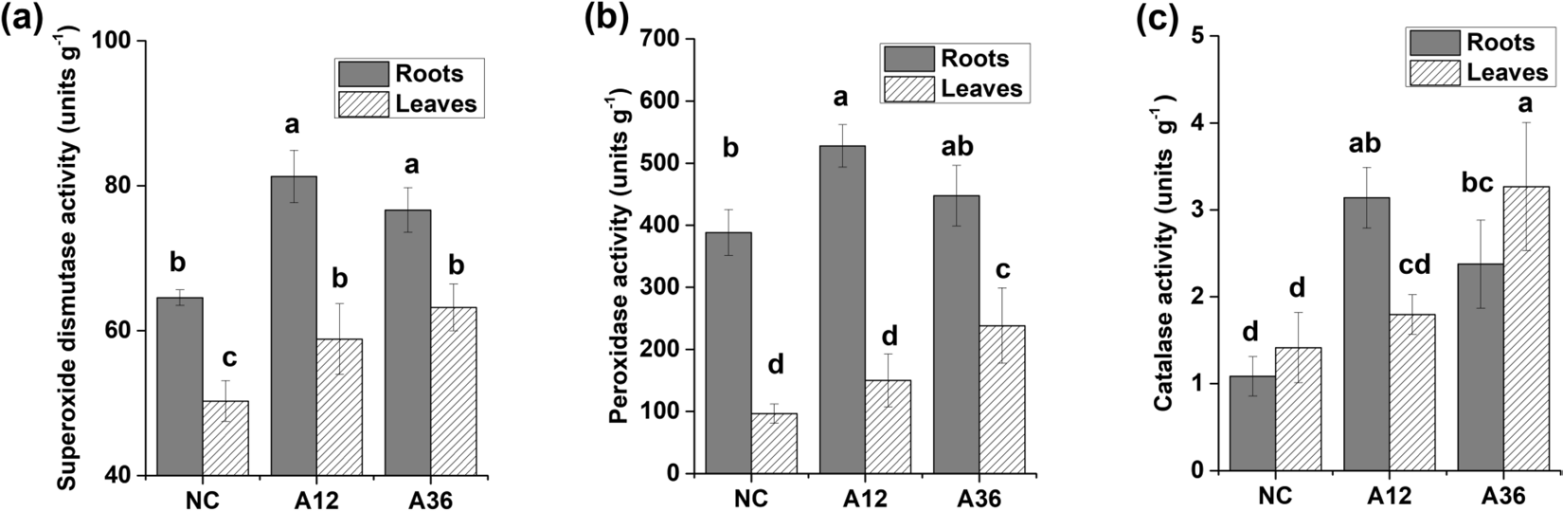
Effects of different levels of NH_4_^+^ on the activity of superoxide dismutase (**a**), peroxidase (**b**) and catalase (**c**) related to reactive oxygen species removal (means ± SDs, n = 5). Different lowercase letters within groups indicate significantly different values (*p* < 0.05).

In response to increased ROS (Supplementary Fig. S2a,b), the activity of antioxidative enzymes was elevated in both the A12 and A36 groups. SOD activity in the roots and leaves under NH_4_^+^ stress was significantly higher than that in the NC group (Fig. 7a). However, POD activity in the leaves was significantly higher in only the A36 group (Fig. 7b). Further, POD activity in the roots initially significantly increased in the A12 group but slightly decreased in the A36 group. Furthermore, CAT activity was also observed to increase in both the roots and leaves of the NH_4_^+^-stressed groups, especially in the roots of the A12 group and the leaves of the A36 group (Fig. 7c). In fact, CAT activity also slightly decreased in the roots of the A36 group compared to the A12 group, although the activity in the A36 group was still significantly higher than that in the NC group. Interestingly, NH_4_^+^ stress provoked the increased expression of many genes in the stress response category, which are related to abiotic and biotic stress (Fig. 2, Supplementary Table S3).

#### Secondary metabolites

**Figure 8:**
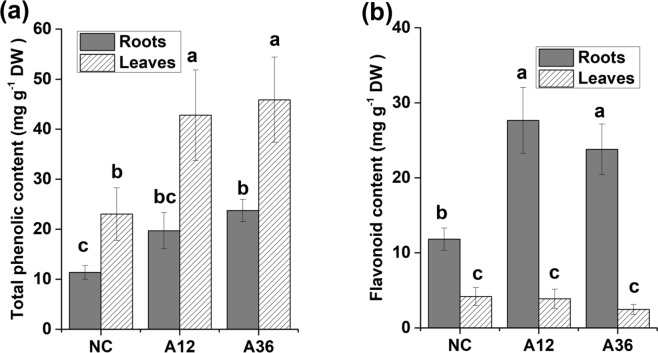
Effect of NH_4_^+^ on the phenolic (**a**) and flavonoid (**b**) contents in *Myriophyllum aquaticum* roots and leaves. The values are the means ± SDs (n = 5). Different letters indicate a significant difference at *p* < 0.05.

With the upregulation of multi-genes involved in phenylpropanoid biosynthesis (Supplementary Table S3), the total phenolic content in leaves was significantly increased when plants were treated with 12 mM NH_4_^+^, although the increase in the content in roots was not significant (Fig. 8a). However, the total phenolic content in both the roots and leaves of the A36 group was significantly higher than that of the NC group. Total phenolics seemed to accumulate primarily in the leaves of all groups, as the content in leaves was always higher than that in roots in the same group. Similarly, the expression of genes in the flavonoid pathway encoding chalcone synthase (CHS), chalcone isomerase (CHI), naringenin 3-dioxygenase (N3D) and flavonol synthase (FLS) was also increased. Due to the increased expression of genes involved in the flavonoid biosynthesis pathway, the flavonoid content also significantly increased in the roots of the A12 and A36 groups by more than 2-fold (Fig. 8b). Interestingly, flavonoids mainly accumulated in the roots of all groups, which was different from the pattern observed for total phenolics. In fact, the flavonoid content in roots was more than ten times higher than that in leaves in the same group, especially when plants were treated with 12 or 36 mM NH_4_^+^.

## Discussion

**Figure 9:**
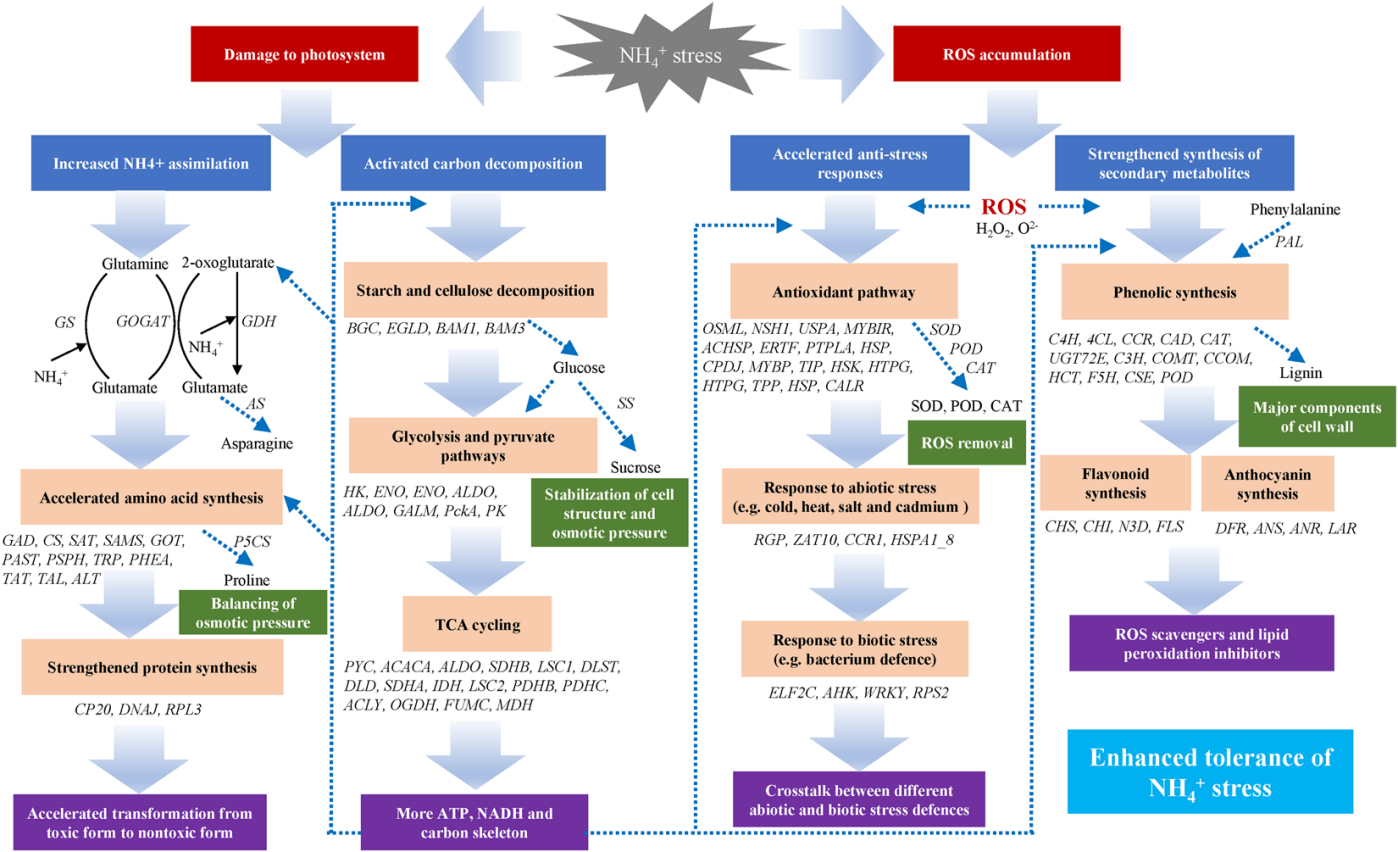
Physiological and genetic regulatory network in *Myriophyllum aquaticum* responding to NH_4_^+^ stress. Significantly regulated genes involved in nitrogen metabolism, carbon metabolism, stress response and secondary metabolism are shown in italics.

Previous studies have proven that *M. aquaticum* is highly tolerant of aquatic environments with high concentrations of NH_4_^+ ^^5,26,27^. However, the physiological and genetic regulations associated with this tolerance are poorly understood. This is the first study on the gene regulation mechanism of high tolerance of NH_4_^+^ toxicity in *M. aquaticum* using RNA-seq with the goal of constructing a physiological and genetic regulatory network for this species (Fig. 9). To the best of our knowledge, these regulations have not previously been systematically investigated in aquatic and wetland plant species.

### Nitrogen metabolism responses to NH_4_^+^ toxicity

In our study, NH_4_^+^ content in leaves is always higher than that in roots under the same NH_4_^+^ concentration (Fig. 1a). Because of the toxic nature of NH_4_^+^, NH_4_^+^ should be assimilated into amino acids in roots once it was absorbed from sediment and then amino acids (Asp, Glu, Asn and Gln) were transported to shoots and leaves via xylem and phloem. However, when plants were treated with ammonium as the sole nitrogen, NH_4_^+^ content in shoots and leaves seemed to rise and could reach concentration in the millimolar range^32,33^. Excess NH_4_^+^ might be loaded into the xylem and transported to leaves via ammonium transporters or passively via aquaporins or nonselective cation and K^+^-specific channels^34–36^. Due to an increase in the NH_4_^+^ content in roots and leaves, many processes connected to nitrogen metabolism were affected. Stimulated by high concentrations of NH_4_^+^, the activity of GS and GOGAT was significantly increased in leaves under NH_4_^+^ stress^3,9^. Surprisingly, GS activity in the roots of *M. aquaticum* maintained a high level even without NH_4_^+^ stress, which indicated that an immediate mechanism of NH_4_^+^ assimilation occurs in *M. aquaticum* roots^31,37^. However, Gln content in leaves was always lower than that in roots under the same NH_4_^+^ concentration, although GS activity in roots was higher than that in leaves. The reason could be that synthesised Gln in roots normally transported to shoots and leaves for storage and signalling, which led to the higher Gln content in leaves than that in roots^36,38,39^. Interestingly, expression of the gene encoding GDH and GDH activity were both significantly increased in both the roots and leaves of both NH_4_^+^-stressed groups, which indicated that GDH might also participate in an important anabolic reaction to scavenge NH_4_^+^ in NH_4_^+^-stressed cells of *M. aquaticum*. This scavenging could be an alternative strategy for transforming NH_4_^+^ into glutamate to avoid NH_4_^+^ damage to plant cells^40,41^. Previous research has shown that AS plays an important role in nitrogen reactivation and can be used to improve nitrogen use efficiency^5,10^. In the present study, NH_4_^+^ stress was found to significantly stimulate AS activity in *M. aquaticum* roots, which indicated that AS is closely associated with the detoxification of NH_4_^+^ in *M. aquaticum*, as reported in a previous study^5^. Thus, GS was the main enzyme responsible for NH_4_^+^ assimilation in *M. aquaticum* under normal concentrations of NH_4_^+^, while many enzymes (such as GDH, GOGAT, AS and GS) were activated and involved in NH_4_^+^ detoxification and scavenging under NH_4_^+^ stress.

Free amino acids play crucial roles in an array of cellular functions, including nitrogen storage, osmotic regulation, and free-radical scavenging under various stresses^12,42^. Several amino acids are also precursors for certain phytohormones that regulate plant growth and development^42^. As a result of NH_4_^+^ stress, the expression of key enzymes involved in Glu, Tyr, histidine, arginine, Ser, threonine and valine synthesis was increased (Supplementary Table S3)^9,12^, and the synthesis of most free amino acids was correspondingly accelerated (Fig. 5). As a large amount of Glu was consumed for Gln synthesis, upregulation of the activity of GDH and GOGAT seemed to lead to the synthesis of more glutamate to guarantee the availability of glutamate for biosynthesis of amino acids. These results suggest that Glu may play a key role in NH_4_^+^ tolerance in *M. aquaticum*, which is consistent with the many known roles of these amino acids^1,9^. Glu is also the precursor of Pro, which serves as an osmoticum in response to different types of stress^42^. Interestingly, we did not find an increase in free Pro content in roots under NH_4_^+^ stress. However, significantly increased Pro content were observed in NH_4_^+^-stressed leaves, which indicated that Pro mainly plays a role in the leaves of *M. aquaticum*. In addition to these functions, different free amino acids are also basic substrates for protein biosynthesis. Thus, *M. aquaticum* might try to avoid NH_4_^+^ toxicity by accelerating the conversion of NH_4_^+^ to free amino acids and subsequently to proteins^2,9^.

### Carbon metabolism responses to NH_4_^+^ toxicity

Chlorosis is one of the main responses of *M. aquaticum* to NH_4_^+^ toxicity^27,43^. The reduced Chl a and Chl b in NH_4_^+^-stressed cells are probably caused by the repression of their biosynthesis. In addition, the coordinated decrease in Chl and Chl-binding proteins under NH_4_^+^ toxicity suggests that the biosynthesis of Chl is coordinated with Chl-binding proteins, which was also observed in a previous study^44,45^. These results clearly indicate that NH_4_^+^ toxicity reduces photosynthetic efficiency, which is in agreement with the observed reduction in the Fv/Fm. Organic acids play a special role in C4 photosynthetic metabolism as intermediate pools of fixed carbon^46,47^. Since PPC is essential for the fixation of CO_2_ into oxaloacetate, its downregulation (Supplementary Table S3) might significantly repress the efficiency of CO_2_ fixation in the C4-dicarboxylic acid pathway^48^ and might lead to the production of more ROS because of excessive energy around the photosystem^43,45^. As a result of the decreased efficiency of carbon and light energy acquisition, *M. aquaticum* seemed to have less carbon and energy for NH_4_^+^ resistance.

Starch and cellulose are the major storage carbohydrates in plants and are most commonly associated with storage organs such as roots, rhizomes, stems and seeds^18,49,50^. A number of studies have shown that many plant species, including several important crop species, can remobilize starch and cellulose reserves to release carbon and energy to resist abiotic or biotic stress^11,12,50^. Hydrolysis of starch to maltose by BAM represents the predominant pathway of starch degradation. In the A36 group, the activity of amylase significantly increased, and the gene encoding BAM3 was upregulated at the same time in roots and leaves. In addition to starch metabolism, cellulose degradation also seemed to be accelerated by the significant upregulation of β-glucosidase activity to generate more glucose. Moreover, the biosynthesis of sucrose was promoted by the upregulation of sucrose synthase. Glucose and sucrose can help stabilize proteins and cell structures, particularly when the stress becomes severe or persists for longer periods^49,50^. These compounds can also act as free-radical scavengers, protecting against oxidation by removing excessive ROS, re-establishing the cellular redox balance^51^ and acting as signalling molecules connected to the ABA-dependent signalling pathway to activate downstream stress-response regulation^11,29,49^.

Accelerated degradation of starch and cellulose releases more glucose, which then enters the glycolysis and pyruvate pathways^11,49^. With many genes involved in glycolysis and pyruvate pathways being upregulated under NH_4_^+^ stress, more acetyl-CoA, the final product of those pathways and the original substrate for the TCA cycle, enters the TCA cycle in the mitochondria^45,52^. Regarding the three key catalytic enzymes in the TCA cycle^37,52^, genes encoding CS and OGDH were significantly upregulated in the NH_4_^+^-stressed root cells of both the A12 and A36 groups, whilst the expression of the isocitrate dehydrogenase (ICDH)-encoding gene was significantly upregulated in the leaves of both the A12 and A36 groups. The TCA cycle can regulate the energy and NADH levels in plant tissues and supply substrates for amino acid synthesis^47^. In response to high concentrations of NH_4_^+^, 2-OG in the TCA cycle furnishes the GS/GOGAT cycle with the carbon skeleton required for amino acid biosynthesis^37,52^. The NH_4_^+^-dependent enhancement of carbon input to the TCA cycle is associated with increased NH_4_^+^ assimilation, which is supported by the higher concentrations of amino acids and proteins observed under excess NH_4_^+^ supply^52,53^. Thus, increased degradation of starch and cellulose, along with induction of glycolysis and the TCA cycle, could provide NH_4_^+^-stressed cells with enough energy and NADH as well as many other substrates for NH_4_^+^ assimilation and amino acid and protein biosynthesis^47,52^.

### ROS removal and secondary metabolisms responses to NH_4_^+^ toxicity

Generally, environmental stresses disrupt internal ROS homeostasis, thereafter leading to oxidative stress and cellular damage in plants^1,13^. In this study, *M. aquaticum* differentially regulated the expression level of genes encoding several representative antioxidant enzymes, such as SOD, POD and CAT, under NH_4_^+^ stress for ROS removal^1^. However, NH_4_^+^-induced inhibition of antioxidative enzyme activities was observed in the A36 group, which indicated a threshold for NH_4_^+^ tolerance in *M. aquaticum*^20,27^. Although redundant ROS damage the nucleic acids, proteins and lipids in NH_4_^+^-stressed cells^54^, some researchers have suggested that ROS accumulation induced by NH_4_^+^ stress is important for defence against a multitude of environmental stimuli, such as pathogens, salinity stress and cadmium-induced oxidative damage^13,54,55^. Our transcriptomic results also showed that NH_4_^+^ stress modulated the expression of many genes involved in abiotic and biotic stress responses (e.g., oxidation, cold, heat, and bacterium defences) (Fig. 2), which also confirms the evidence that NH_4_^+^-triggered antioxidant defence might be a universal strategy for adaptive plant responses to multiple environmental stimuli^14,30,55,56^.

Phenols perform a wide range of functions to mitigate the effect of oxidative stress by acting as ROS scavengers and lipid peroxidation inhibitors, thereby protecting important cellular components such as photosynthetic apparatuses^15,31^. In this study, the phenolic and flavonoid contents were both observed to increase under NH_4_^+^ stress and mainly accumulated in different plant tissues. KEGG analysis also revealed that most of the genes involved in the phenylpropanoid biosynthesis pathway were upregulated under NH_4_^+^ stress (Supplementary Table S3), especially the genes encoding the key enzymes that catalyse multiple reactions in this pathway, such as 4-coumarate-CoA ligase (4CL), cinnamoyl-CoA reductase (CCR) and cinnamyl-alcohol dehydrogenase (CAD), which was also reported in previous studies^15,16^. The end products of the phenylpropanoid biosynthesis pathway are different kinds of lignin originating from Phe^10^. Flavonoids may constitute a ‘secondary’ antioxidant system in response to severe stress to complement the role of antioxidant enzymes in combating that stress^10,16,17^. Thus, flavonoids accumulated in *M. aquaticum* tissues may also act as osmolytes to protect plants from NH_4_^+^ stress damage^17^. Interestingly, genes for anthocyanin biosynthesis, such as dihydroflavonol 4-reductase (DFR), anthocyanidin synthase (ANS), anthocyanidin reductase (ANR) and leucoanthocyanidin reductase (LAR), were also upregulated under NH_4_^+^ stress (Supplementary Table S3). The demonstration of the accelerated synthesis of phenylpropanoids and their derivatives such as phenolics, flavonoids, anthocyanins and lignin in NH_4_^+^-stressed *M. aquaticum* further emphasized the importance of these secondary compounds for the adaptation of *M. aquaticum* to high-NH_4_^+^ environments.

## Conclusions

The present work indicated that a complex physiological and genetic regulatory network including nitrogen metabolism, carbon metabolism, abiotic stress response and secondary metabolism at the root and leaf levels was involved in NH_4_^+^ resistance in *M. aquaticum*. The synthesis of amino acids and proteins was accelerated to assimilate NH_4_^+^ in plant tissues. Furthermore, starch and cellulose were decomposed for greater supplies of carbon and energy. Antioxidative enzyme activity and the secondary metabolite content were also significantly elevated to remove redundant ROS.

## Methods

### Experimental design

This study was conducted in a greenhouse at a stable temperature (25–30 °C) and under a 12/12 h dark/light photoperiod at the Research Center for Eco-Environmental Sciences, Chinese Academy of Sciences, Beijing. Polyethylene plastic containers (0.5 m × 0.4 m × 0.4 m) were filled with approximately 10 cm of sterile quartz sand (Φ = 1–3 mm). *M. aquaticum* seedlings with a uniform length of 50 ± 3 cm were planted in plastic containers. Before the experiments, plants were cultured for two weeks in 50% modified Hoagland solution, as described previously^27^. After a two-week preincubation, plants were transplanted into plastic containers. The culture solution in each container consisted of 25 L 50% modified Hoagland solution amended with various concentrations of NH_4_Cl. Plants treated with 12 mM NH_4_Cl were defined as the A12 group, whilst plants treated with 36 mM NH_4_Cl were defined as the A36 group. A negative control (NC) group was treated with 1 mM NH_4_Cl. Each group contained five containers as replicates, and each container housed 30 seedlings. The pH of the culture solution in each container was adjusted to 6.0 ± 0.1 with 1 M KOH. The culture solution was exchanged every 2 days to maintain the pH and nutrient concentrations. After 14 days of cultivation, plants in each container were harvested. Root and leaf samples rinsed with distilled water were ground with liquid nitrogen and fully mixed^12,15^. Part of the mixture was used for tissue and enzyme analysis, whilst the remainder was used for RNA extraction.

### Phenotype analysis

To determine plant growth, plants were harvested on day 14 and then measured for growth rate as previously described^25^. Fv/Fm was measured using an LI-6400 portable photosynthesis system (LI-COR Inc., Lincoln, Nebraska, USA), whilst the Chl a (Chl a), Chl b (Chl b) and total Car contents in plant leaves were measured with a spectrophotometer (DR6000, HACH, USA) after extraction with 80% acetone^57^. The NH_4_^+^ content in plant tissues was measured by using the method described^5^ and expressed as µg g^−1^ FW. The level of lipid peroxidation in plant leaves was evaluated by determining the MDA content using the thiobarbituric acid test^58^. The H_2_O_2_ content was determined according to the method^59^ by analysing the titanium-hydroperoxide complex production at 410 nm. The O^2-^ content was measured by the oxidation of hydroxylamine at 530 nm as described previously^60^. The amounts of Gln, Asn and free amino acids were determined by an L-8900 high-speed amino acid analyser (Hitachi, Japan)^15^. Protein was extracted and determined by using the method described^61^. The soluble carbohydrate, glucose, fructose and sucrose contents in plant tissues were determined as described previously^10^. Total phenolics and flavonoids were expressed as µg g^−1^ dry weight (DW) using gallic acid and rutin as standards, respectively^62,63^.

### Enzyme activity analysis

GS activity was expressed as nmol γ-GHA min^−1^ g^−1^ FW at 25 °C and tested at 540 nm, whilst GDH activity was expressed as nmol NADH consumption min^−1^ g^−1^ FW at 25 °C and monitored spectrophotometrically at 340 nm^61^. GOGAT activity was determined by using the method^48^. AS activity was expressed as nmol NH_4_^+^ consumption min^−1^ g^−1^ FW at 25 °C and determined with a spectrophotometer^64^. Total amylase activity was determined as the speed of maltose production measured at 540 nm^65,66^. β-glucosidase activity was expressed as nmol p-nitrophenol min^−1^ g^−1^ FW at 25 °C and tested at 400 nm^67^. The SOD and POD activities were measured by the method described previously^27^. CAT activity was computed by calculating the speed of H_2_O_2_ decomposition^68^. One unit of CAT activity was defined as the amount of 1 nmol H_2_O_2_ decomposition in one minute.

### RNA extraction and library construction

Root and leaf samples were fully ground with liquid nitrogen, and 200 mg sample was used for total RNA extraction and purified with an EASYspin Plus Complex Plant RNA Kit (Aidlab Biotech, Beijing, China). Three biological replicates of each group were used for RNA-seq analysis. The NEBNext® Ultra™ RNA Library Prep Kit for Illumina® (#E7530L, NEB, USA) was used for sequencing library construction^46^.

### Sequencing, assembly and annotation of the transcriptome

After the index-coded samples were clustered, the libraries were sequenced on a HiSeq X Ten platform, and 150 bp paired-end reads were generated. The raw RNA-seq reads were deposited in the National Center for Biotechnology Information (NCBI) Sequence Read Archive (BioProject accession no. PRJNA497710). Raw reads were processed with Perl scripts to ensure the quality of data used for further analysis. Trinity software was used for de novo assembly^46^. Transcripts and predicted peptides were annotated by the NR, Swiss-Prot, Pfam, UniProt and eggNOG databases.

### Differentially expressed genes and enrichment analysis

HTSeq v0.6.0 was used to count reads for each gene in each sample, and the reads per kilobase per million mapped reads (RPKM) were calculated to estimate the expression level of genes in each sample^69^. An estimated false discovery rate (FDR) was assigned to each gene (RPKM ≥ 1) and adjusted with the Benjamini and Hochberg approach to control the FDR^70^. Genes with an FDR ≤ 0.05 and an |log2 ratio| ≥ 1 were identified as DEGs. GO and KEGG enrichment of DEGs was implemented to draw pathway maps of the molecular interaction and reaction networks^7^.

### Gene expression validation

To further evaluate the reliability of the RNA-seq results, eighteen genes of *M. aquaticum* were selected for qRT-PCR to assess their expression in response to NH_4_^+^ stress. The β-actin gene was used as an internal control. The relative expression levels were calculated as previously described^71^.

### Data analysis

IBM SPSS 20.0 software (IBM Corp.) was used for data analysis. One-way analysis of variance with Tukey’s test was applied to identify significant differences between groups and between tissues (*p* < 0.05). A Spearman two-tailed test was used to identify significant correlations (*p* < 0.05) between growth characteristics and NH_4_^+^ content in the plant samples.

## Supplementary information

Supplementary Figures

Supplementary Table 1

Supplementary Table 2

Supplementary Table 3

Supplementary Table 4
