# Supplementary material for: Complex regulatory network allows Myriophyllum aquaticum to thrive under high-concentration ammonia toxicity
Source: Sci Rep. 2019 Mar 18;9:4801. doi: 10.1038/s41598-019-41236-8 (PMC6423053; doi:10.1038/s41598-019-41236-8)
Supplement: Supplementary file 1 — Supplementary Figures [file 41598_2019_41236_MOESM1_ESM.pdf]

***Scientific Reports* Supplementary Information**

Complex regulatory network allows *Myriophyllum aquaticum* to thrive under high-concentration ammonia toxicity

Rui Wang[1, 2,†], Shengjun Xu[1, 2,†], Haishu Sun[1, 2], Shugeng Feng[1, 2], Cancan Jiang[1, 2], Sining Zhou[1, 2], Shimin Wu[1, 2], Guoqiang Zhuang[1, 2], Baodong Chen[2, 3], Xuliang Zhuang[1, 2*] and Zhihui Bai[1, 2*]

*Corresponding author: X.Z. (email: xlzhuang@rcees.ac.cn) and Z.B. (email: zhbai@rcees.ac.cn)

The following Supplementary Information is available for this article:

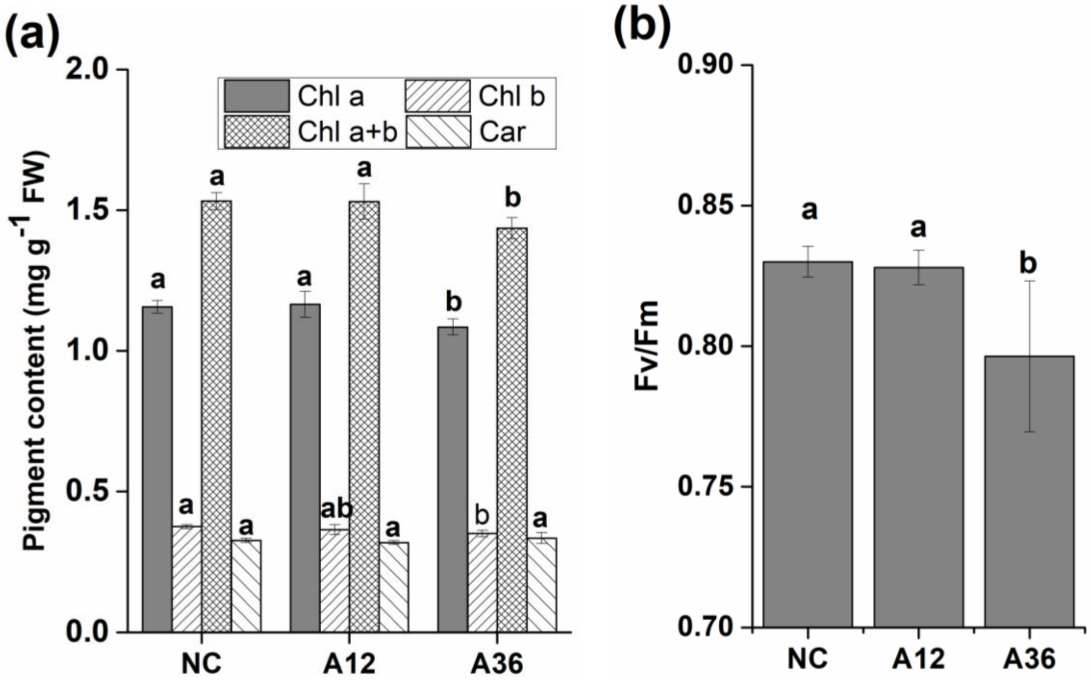

Supplementary Fig. S1 Change in photosynthetic pigment content and maximum quantum yield of PSII (Fv/Fm) under NH_4^+ stress. (a) Photosynthetic pigment content. (b) Fv/Fm. Error bars represent the means ± SDs (n = 5). Different letters indicate a significant difference at $p < 0.05$.

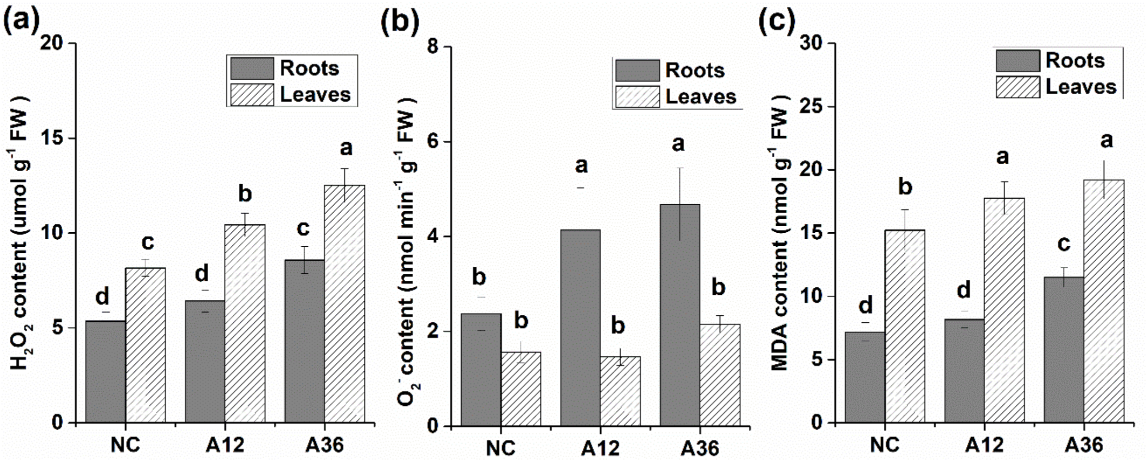

Supplementary Fig. S2 Reactive oxygen species and malondialdehyde (MDA) content. (a) H_2O_2 content. (b) O^{2-} content. (c) MDA content. Values (means ± SDs) were determined from five biological replicates (n = 5). Different letters above the bars indicate a significant difference at $p < 0.05$.

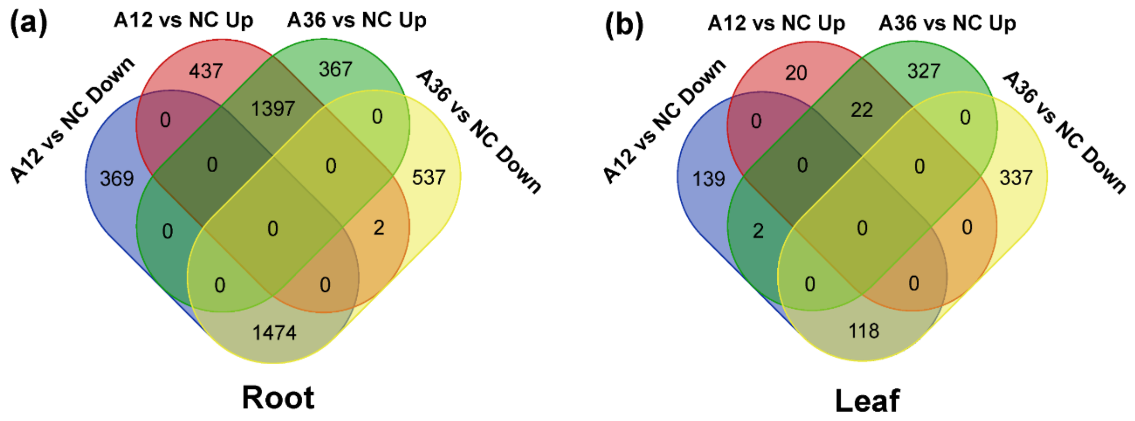

Supplementary Fig. S3 Venn diagram of overlapping significant differentially expressed genes (DEGs) in roots (a) and leaves (b) of *Myriophyllum aquaticum* when exposed to 1 mM (NC), 12 mM (A12) or 36 mM (A36) NH_4^+.

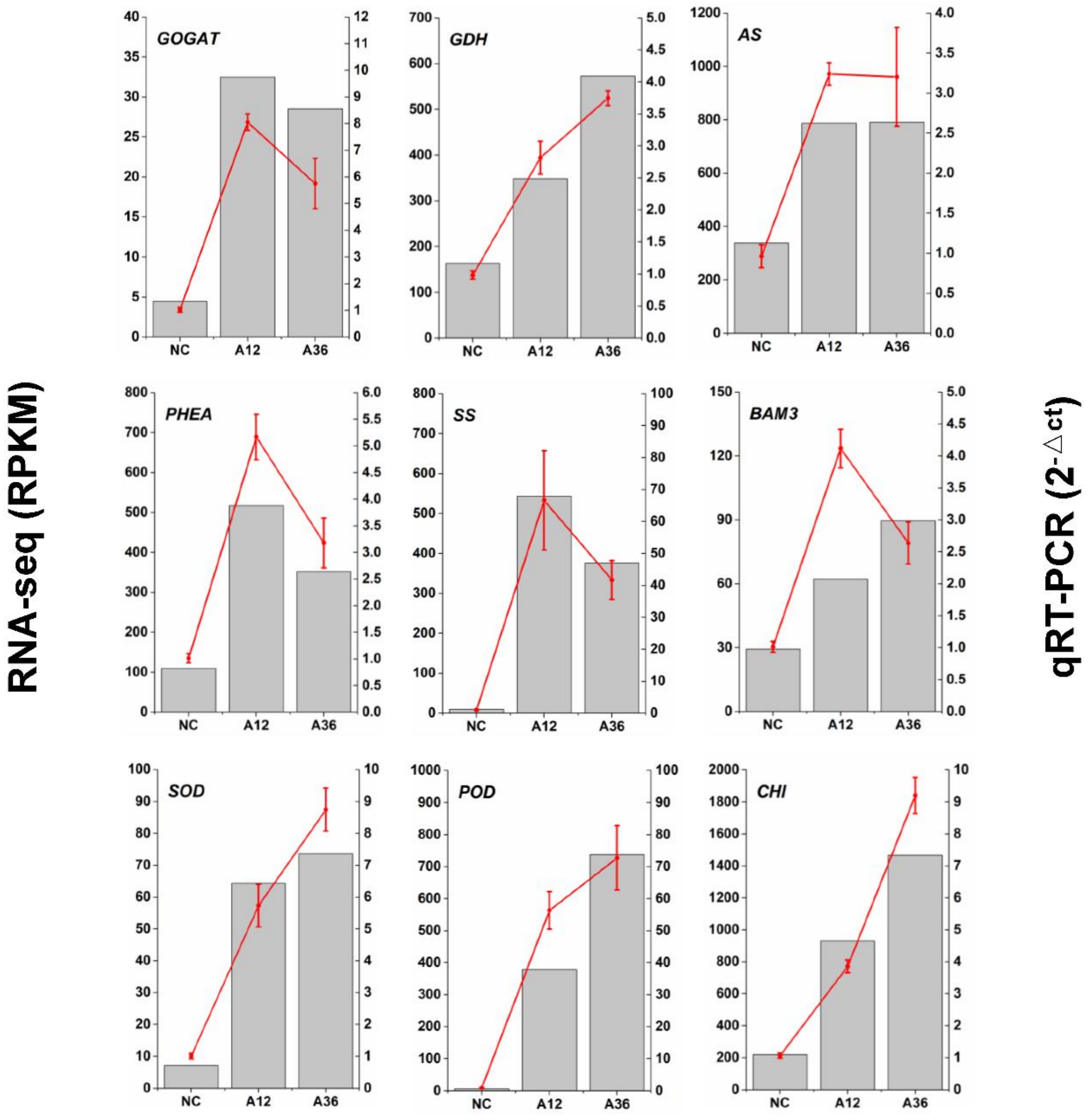

Supplementary Fig. S4 qRT-PCR validation of differentially expressed genes in *Myriophyllum aquaticum* roots under NH_4^+ stress. The axes represent the reads per kilobase per million mapped reads (RPKM) obtained for each gene in the preliminary RNA-seq analysis (grey bars) and the fold change in expression (red lines and circles) observed in replicated qRT-PCR analyses. Values (means ± SDs) were determined from three biological replicates (n = 3).

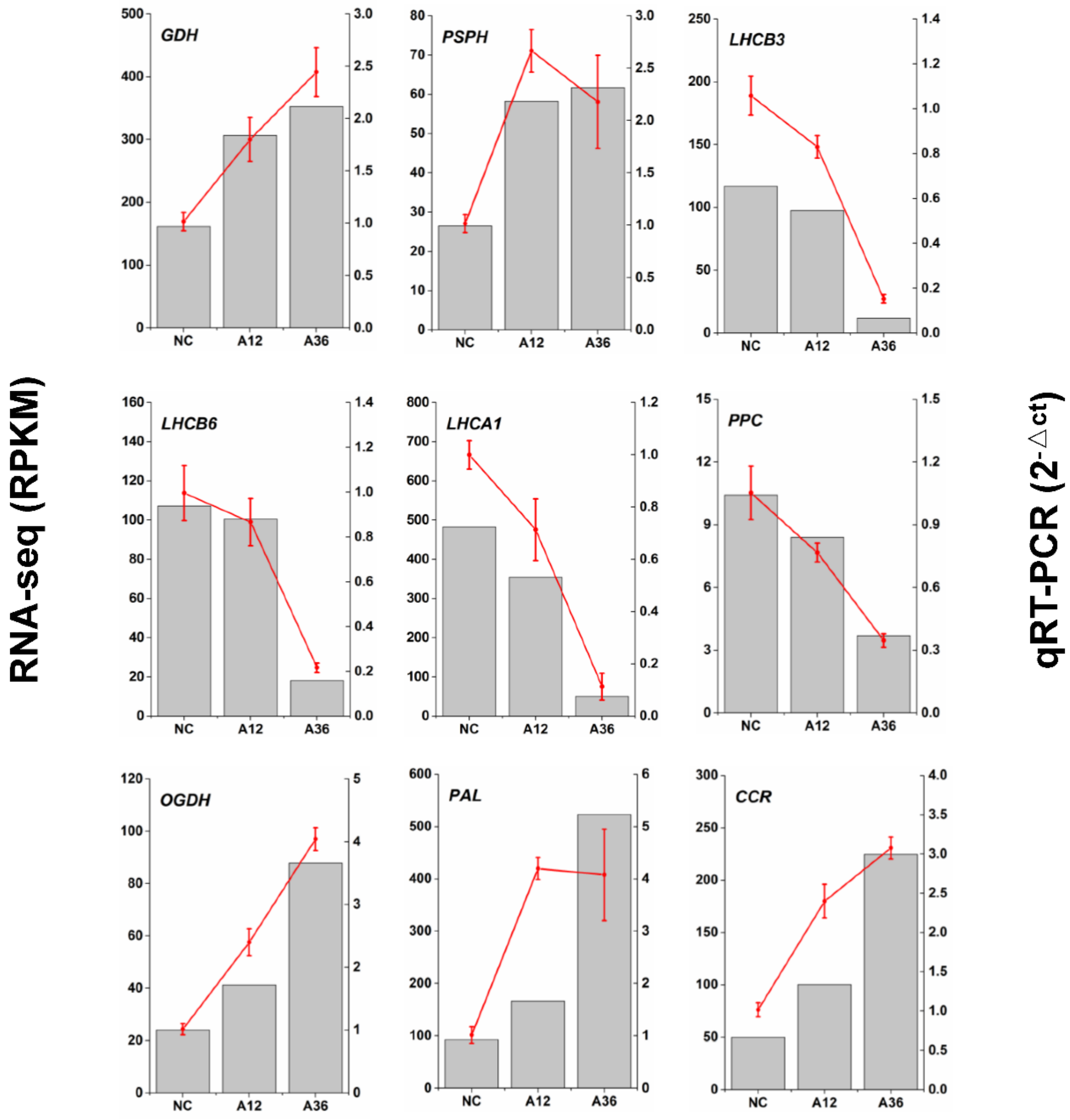

Supplementary Fig. S5 qRT-PCR validation of differentially expressed genes in *Myriophyllum aquaticum* leaves under NH_4^+ stress. The axes represent the reads per kilobase per million mapped reads (RPKM) observed in RNA-seq analysis (grey bars) and the fold change in expression (red lines and circles) observed in qRT-PCR analyses. Values (means ± SDs) were determined from three biological replicates (n = 3).

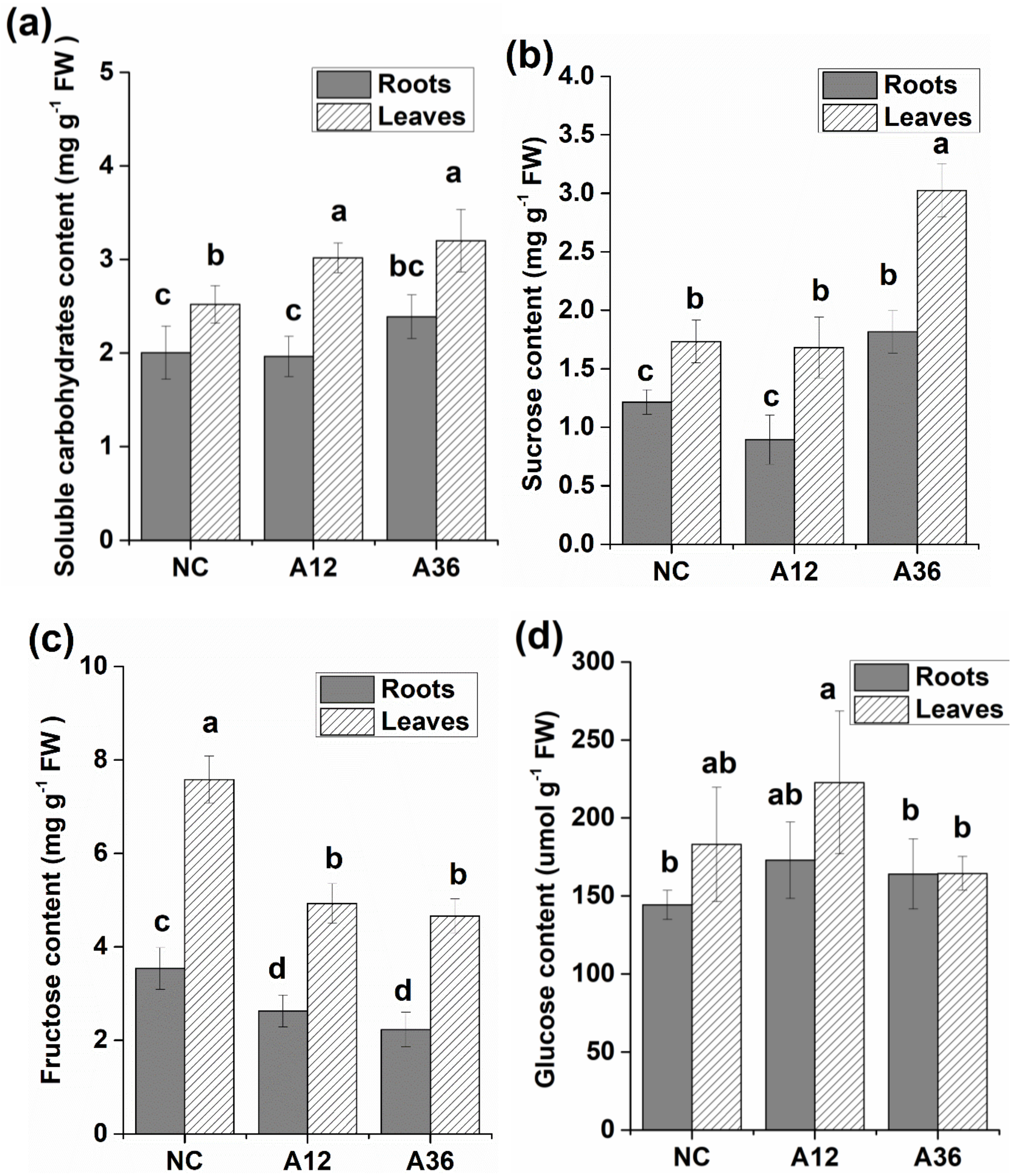

Supplementary Fig. S6 Effect of NH_4^+ toxicity on soluble carbohydrates (a), sucrose (b), fructose (c) and glucose content (d) in *Myriophyllum aquaticum* roots and leaves. Error bars represent the means ± SDs (n = 5). Different letters indicate a significant difference at $p < 0.05$.
